# Malondialdehyde as an Important Key Factor of Molecular Mechanisms of Vascular Wall Damage under Heart Diseases Development

**DOI:** 10.3390/ijms24010128

**Published:** 2022-12-21

**Authors:** Vadim Z. Lankin, Alla K. Tikhaze, Arthur M. Melkumyants

**Affiliations:** Department for Free Radical Research, National Medical Research Center of Cardiology, Russian Ministry of Health, 121552 Moscow, Russia

**Keywords:** atherosclerosis, free radicals, lipid peroxidation, malondialdehyde, LDL, endothelial glycocalyx

## Abstract

This mini review is devoted to a specific issue: the role of malondialdehyde (MDA)—a secondary product of free radical lipid peroxidation—in the molecular mechanisms of the formation of primary atherosclerotic vascular wall lesions. The principal difference between this review and the available literature is that it discusses in detail the important role in atherogenesis not of “oxidized” LDL (i.e., LDL particles containing lipohydroperoxides), but of LDL particles chemically modified by the natural low-molecular weight dicarbonyl MDA. To confirm this, we consider the data obtained by us earlier, indicating that “atherogenic” are not LDL oxidized as a result of free radical lipoperoxidation and containing lipohydroperoxy derivatives of phospholipids in the outer layer of particles, but LDL whose apoprotein B-100 has been modified due to the chemical reaction of terminal lysine residue amino groups of the apoB-100 with the aldehyde groups of the MDA (Maillard reaction). In addition, we present our original data proving that MDA injures endothelial glycocalyx that suppress the ability of the endothelium to control arterial tone according to changes in wall shear stress. In summary, this mini review for the first time exhaustively discloses the key role of MDA in atherogenesis.

## 1. Introduction: Atherosclerosis as a Free Radical Disease

The assumption that spontaneous free radical processes can play a major role in atherosclerosis etiology and progression was made back in the late 1950s [1]. However, only two decades later we managed to experimentally prove a considerable increase in the content of lipohydroperoxides (LOOH), being the primary products of free radical peroxidation, in the blood of patients with atherosclerosis and in the vascular wall damaged with experimental atherosclerosis [2,3]. We also revealed that in the blood of patients with atherosclerosis, the activity of Se-containing glutathione peroxidase (GSH-Px) in the red blood cells, catalyzing LOOH reduction, is below normal. In case of atherosclerotic-induced injury of a vascular wall, the activity of GSH-Px and Cu,Zn-superoxide dismutase (Cu,Zn-SOD) was significantly decreased, correlating well with the severity of injury [2,3]. The obtained results allowed us to establish an imbalance between the formation and utilization of free radical peroxidation products in tissues in cases of atherosclerosis [2,3]. This imbalance was later termed an oxidative stress by H. Sies [4]. It goes without saying that preaterosclerotic (lipoidosis) damage of vascular walls could be induced by disruptions to the lipid transport system, which is provided by the low-density lipoproteins (LDL) being natural nanoparticles from human blood plasma. Indeed, chemical modification of the apoprotein B-100 of LDL makes them more “atherogenic” [5], i.e., capable of being captured by scavenger receptors of macrophages in the vascular wall [5]. Many authors have suggested that the atherogenic modification of LDL particles can be brought about by a more intensive spontaneous free radical oxidation of the outer unsaturated phospholipid layer of LDL with LOOH formation [2,3]. Evidence on the increased concentration of oxidized LDL in cases of atherosclerosis has been numerously reported [2,3].

## 2. Stages of Free Radical Peroxidation of Unsaturated Phospholipids in LDL Particles: MDA Accumulation Mechanism

Free radical oxidation of the outer phospholipid layer of LDL particles can be initiated by reactive oxygen species (ROS) formed in the process of oxidative stress during atherogenesis [2,3]:

O_2_ ^•−^ → H_2_O_2_ → HO^•^

According to the theory of liquid phase oxidation of hydrocarbons, free radical peroxidation of LDL should have two stages: under the attack by ROS, first, the primary products of free radical peroxidation (LOOH) are produced from unsaturated LDL phospholipids (LH) [2,3]:

LH + HO^•^ → H_2_O + L^•^

L^•^ + O_2_ → LO_2_^•^

LO_2_^•^ + LH → LOOH + L^•^

Then, the unstable LOOH undergo destruction with formation of alkoxyl radicals (LO^•^), which leads to further oxidation of the lipid substrate (LH) [2,3]:

LOOH → OH^−^ + LO^•^

LH + LO^•^ → LOH + L^•^

**Figure 1:**
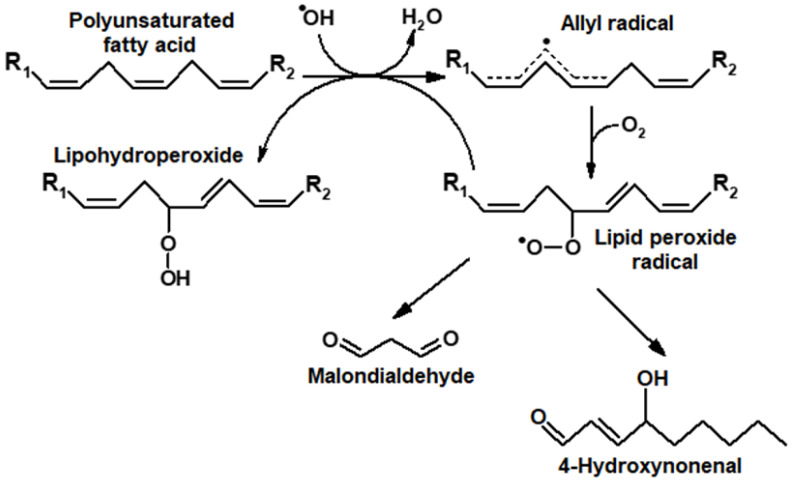
Formation of primary (LOOH) and secondary products (carbonyl compounds) during the induction of oxidative stress by oxygen radicals (ROS).

The oxidative breakdown of LOOH could also accompany this process, with secondary products being formed, namely 4-hydroxynonenal and low-molecular-weight dicarbonyl malondialdehyde (MDA) [2] (Figure 1).

**Figure 2:**
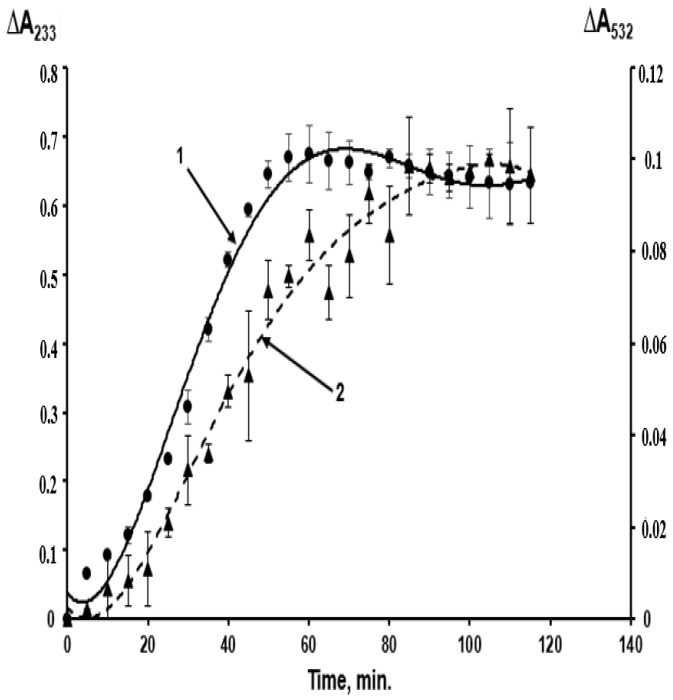
Kinetics of free radical peroxidation of phospholipids of the outer layer of LDL particles isolated from blood plasma of a healthy donor initiated by copper ions, studied by the accumulation of primary—LOOH (curve 1, left scale — _Δ_A_233_) and secondary—MDA (curve 2, right scale — _Δ_A_532_) products of lipoperoxidation. The determination of lipoperoxides and MDA was carried out as described in [2].

Unfortunately, these oxidation stages were largely dismissed in studies on LDL peroxidation in case of atherosclerosis [2,3]. Hence, LDL oxidized by free radicals under the impact of various initiating agents were characterized as “oxidized” LDL. Figure 2 shows the kinetics of LDL lipid peroxidation in a standard system when oxidation of LDL particles is initiated by copper ions.

**Figure 3:**
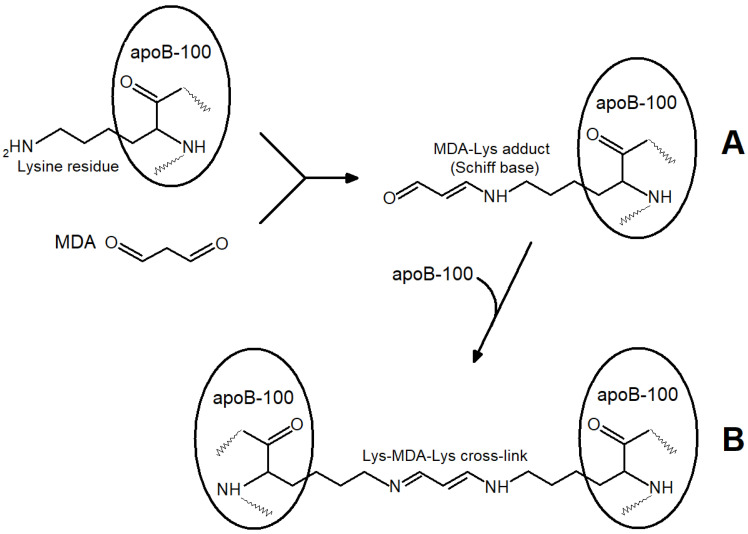
Reactions of end lysine residues of apoprotein B-100 LDL particles with MDA aldehyde groups: (**A**)— Maillard reaction: formation of MDA-lysine adduct of apoprotein B-100 LDL (Schiff base); (**B**)—participation of second MDA aldehyde group in formation of intermolecular cross-linking between two LDL particles.

Primary and secondary products (MDA) are formed almost simultaneously, and the “oxidized” LDL contain a considerable amount of MDA, yielding the MDA-modified LDL (Figure 2). A combination of primary and secondary lipoperoxidation products is formed when any initiating agents are used in in vitro models (ions of metals with variable valency, azo-initiators, hydroperoxides, O_2_^•−^ generated by polymorphonuclear leukocytes, UV radiation, etc.) [3]. When the effects of oxidative stress are studied in vivo, researchers also deal with an uncontrolled combination of LOOH-containing and MDA-modified LDL, implicating that there are not only “oxidized” LDL in this combination. The latter term used in some works [6,7,8] is to be substituted with a more accurate “oxidatively modified LDL”. This combination may also account for considerable inconsistencies between the data obtained using in vitro and in vivo models [9,10] (as in these works, the concentration of free radical oxidation products is usually not determined accurately). MDA accumulation may lead to the following biological effects: aldehyde groups of carbonyls can easily react with the end amino groups of proteins via the Maillard reaction, forming intra- (Figure 3A) and intermolecular (Figure 3B) cross-links in their molecules [11].

The reaction of the aldehyde group of MDA with the terminal lysine residue of apoprotein B-100 can alter the molecular conformation of the apoprotein and the whole LDL particle (Figure 3A). The subsequent interaction of MDA with the lysine residue of apoprotein B-100 of the second LDL particle leads to a cross-link between two LDL particles (Figure 3B) and ultimately to a more significant modification where LDL atherogenicity may elevate. Changes in electrophoretic patterns and light scattering fluctuations point to larger LDL formations produced following the interaction of LDL particles with MDA [12].

**Figure 4:**
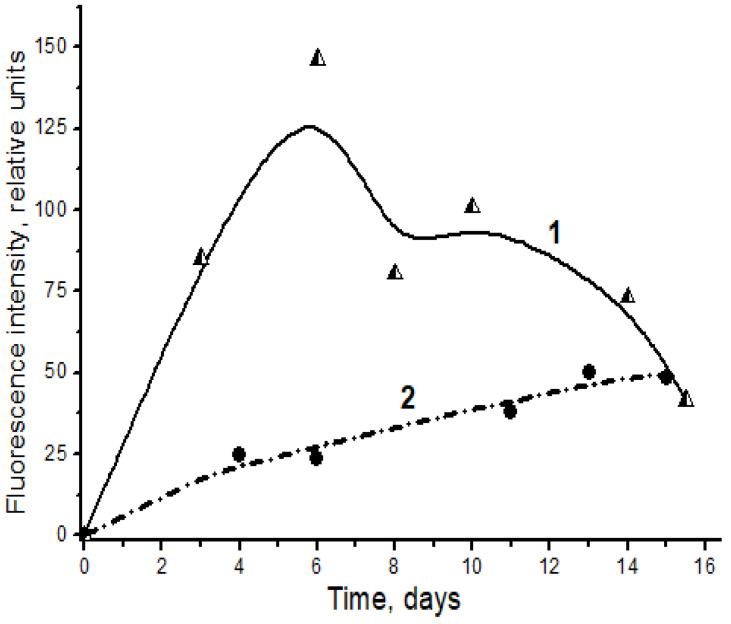
Spontaneous formation of fluorescent Schiff bases in the interaction of l-lysine with MDA (1, triangles) and 4-hydroxynonenal (2, circles). Changes in fluorescence intensity (excitation wavelength λmax = 350 nm) of products of l-lysine (20 mM) interaction with aldehydes (20 mM) during dark incubation at 37 °C in isotonic K,Na-phosphate buffer (pH 7.2) containing 0.02% sodium azide for prevention of bacterial growth. The figure presents mean results of three determinations; the fluorescence of a standard solution of dehydrated quinine sulfate (0.01 μg/mL in 0.05 M H_2_SO_4_) is taken as unit of fluorescence intensity.

Figure 4 shows the kinetics of spontaneous accumulation of fluorescent products in a reaction of l-lysine with carbonyl compounds (4-hydroxynonenal and MDA), formed as secondary products of lipoperoxidation. Clearly, the reaction with MDA is much more effective than the similar reaction with the monoaldehyde 4-hydroxynonenal (Figure 4).

It is obvious that the MDA-modified apoprotein B-100 of LDL should induce conformation changes in the apoprotein structure, which can be recognized by scavenger receptors [13]. However, LOOH acyls formed in LDL phospholipids should also transform their physical properties (microviscosity and polarity), which may further lead to conformation changes of the outer phospholipid layer of particles [14] and, consequently, changes in LDL receptor properties.

## 3. Which LDL Are Atherogenic: LOOH-Containing (“Oxidized”) or MDA-Modified Ones?

**Figure 5:**
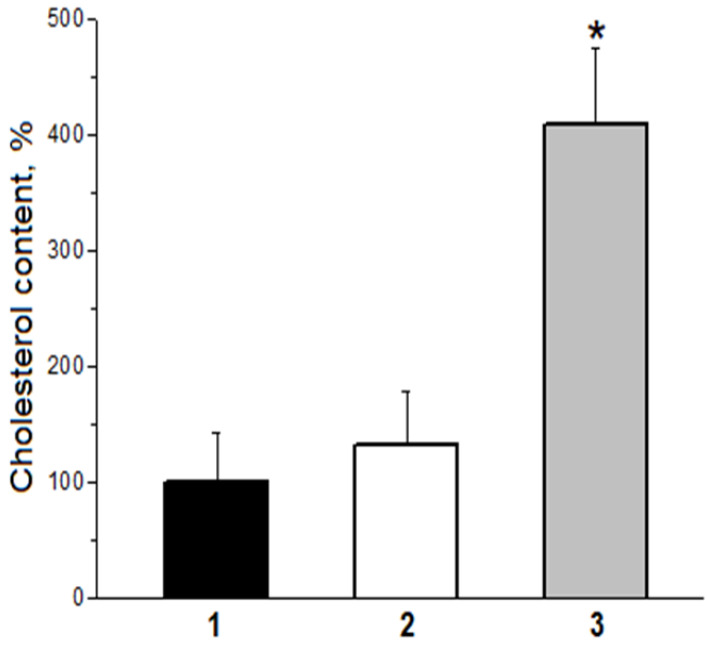
Efficiency of capture by cultured human macrophages of LDL particles modified by primary and secondary products of free radical lipid oxidation. 1—initial native (non-oxidized) LDL; 2—LDL oxidized by animal C-15 LOX (true oxidized, LOOH-containing LDL); 3—MDA-modified LDL (apoB-100—100 µg/mL; * *p* < 0.05).

It was established that MDA-modified LDL particles (MDA-LDL) are effectively captured by the vascular wall macrophages with scavenger receptors and are accumulated in their lipid vacuoles [13,15]. Therefore, MDA-LDL are atherogenic because macrophages ingesting them are transformed into the so-called foam cells laden with lipids, which form lipoidosis zones, the primary pre-aterosclerotic damage to vascular walls [2,3]. Regarding the “oxidized” (acylhydroperoxide-containing) LDL (LOOH-LDL), it is hard to determine their atherogenicity because obtaining LOOH-LDL without MDA-LDL content is fairly impossible (Figure 2). To resolve this problem, we used a homogeneous preparation of C-15 animal rabbit reticulocyte lipoxygenase (C-15 LOX) as a tool [2,16]. Distinct from plant C-15 lipoxygenase (soya been lipoxigenase), which oxidizes the non-esterified (“free”) polyene fatty acids with LOOH formation [2,16], this enzyme catalyzes LOOH formation in acyls of esterified fatty acids, including phospholipids and cholesteryl esters [2,16]. This allowed us to obtain the following from the same sample of isolated native LDL taken from a donor: LOOH-LDL without MDA-LDL content (due to oxidation of LDL by C-15 animal LOX), and MDA-LDL without LOOH-LDL (by chemical modification of native LDL when they were incubated with MDA) [14]. We found out that real “oxidized” LDL (containing LOOH derivatives of phospholipids in the outer layer of a particle) are captured by the cultivated macrophages as effectively as native (non-oxidized) LDL, while MDA-modified LDL demonstrate an extremely effective scavenger receptor binding [15] (Figure 5).

These data laid the foundation for our theory, giving the major role in inducing the atherosclerotic vascular wall damage to the MDA-modified LDL rather than to the “oxidized” ones [3,17]. Moreover, representative epidemiological studies revealed that the most cholesterol-rich LDL particles are also MDA-modified significantly more often (i.e., they are the most atherogenic ones) [18]. An increased level of MDA-LDL was evidenced to be characteristic of patients with certain mutations of apoprotein B-100. This means that MDA-LDL accumulation can be genetically determined [19].

Cu, Zn-SOD and GSH-Px molecules are modified in case of MDA accumulation during atherogenesis, which is akin to apoprotein B-100 [2,3]. Along with this, their activity in tissues is inhibited due to conformational changes of the active center structure [20]. Consequently, MDA accumulation during atherogenesis not only leads to an intensive atherogenic MDA-LDL formation, but also inhibits key antioxidant enzymes, which should promote oxidative stress progression [3,17].

## 4. Role of MDA-Modified LDL in Endothelial Dysfunction

**Figure 6:**
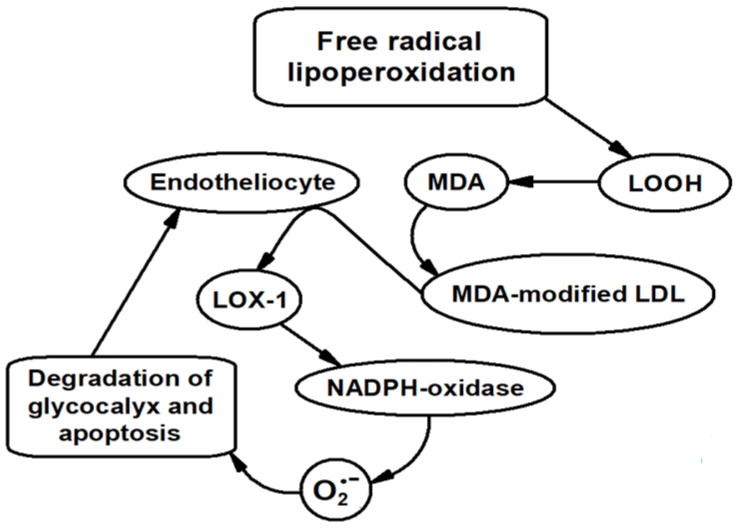
Scheme illustrating the sequence of processes involving MDA leading to endothelial dysfunction.

Aggressive MDA can not only modify LDL apoprotein and other proteins, but also dramatically alter cell membrane properties, including endotheliocyte biomembranes [12,21]. Incubating the cultured endotheliocytes with MDA is accompanied by the increasing stiffness of outer membranes of these cells [12]. In this case, the outer endotheliocyte membrane becomes more permeable to low-molecular-weight substances [21]. In recent years, the oxidatively modified LDL (in our opinion, MDA-LDL) has been discovered to play a major role in endothelial dysfunction [22,23,24,25,26]. It is assumed that oxidatively modified LDL induce the expression of LOX-1 scavenger receptor and NADPH oxidase on the endotheliocyte membrane. NADPH oxidase generates a superoxide anion radical leading to endotheliocyte damage [25,26]. Finally, endothelial dysfunction develops, and endotheliocyte apoptosis is stimulated [25,26]. Our preliminary experiments showed that cultured human umbilical vein endothelial cells (HUVECs) in the presence of MDA-LDL indeed leads to a strong expression of the LOX-1 scavenger receptor and NADPH oxidase (increase in protein expression which is much higher than in the case of cultured cells without MDA-LDL) [17,27]. Therefore, the initial stages of endothelial dysfunction, which is an essential process in atherogenesis, are directly related to formation of oxidatively modified LDL (most probably, MDA-modified ones). Superoxide-dependent endotheliocyte membrane damage probably makes the vascular wall more permeable to MDA-LDL, causing formation of “foam cells” during atherogenesis [2,3,17]. The sequence of the above processes is schematically illustrated in Figure 6.

It should be noted that the enzyme antioxidant system of endotheliocytes is represented by a special type of enzymes, peroxiredoxins [28], which, like Cu,Zn-SOD and GSH-Px, are very sensitive to the inhibiting effect of MDA [29]. It seems absolutely natural that MDA-dependent suppression of peroxiredoxins activity considerably weakens the antiradical protection of endotheliocytes, promoting their damage and endothelial dysfunction progression. Overall, the above data allow us to assume that MDA accumulation and MDA-LDL formation are key factors in processes essential for inducing atherogenesis and endothelial dysfunction.

## 5. Impact of Free Radical Peroxidation of LDL and MDA Accumulation on Glycocalyx Preservation

Two decades of recent studies showed that endothelial dysfunction is preceded by a lesion to the endothelial glycocalyx (EG), a layer of macromolecules produced by endotheliocytes and facing the vascular lumen. The role of this protective layer in the development of endothelial dysfunction is marked in Figure 6. The structure of endothelial cells covering the inner face of the vascular wall is similar to shrubs composed of plasmalemmal-anchored glycosaminoglycans, proteoglycans, glycoproteins, and glycolipids [30,31,32].

EG protects the endothelial cells against detrimental influences, whereas a lesion to EG results in elevation of vascular wall permeability [33,34], rapid progress of atherosclerosis, and functional loss of the potency to regulate the tone of vascular smooth muscles in response to the changes in shear stress affecting the vascular wall [34,35]. These facts permit us to hypothesize that damage to the glycocalyx is the first step provoking the development of atherosclerotic lesions to the blood vessels [36].

Numerous studies showed that the leading factor responsible for damage to the glycocalyx and abnormal endothelial performance in atherosclerosis is the oxidative stress [37,38,39,40,41] accompanied by the ROS overproduction and lipid peroxidation with formation of LOOH and the secondary oxidative product, MDA. The latter is amply produced during this free radical oxidation [42]; it is noteworthy that this agent is a structural analog to glutaraldehyde. This similarity is important because our previous studies on circulatory isolated feline conduit arteries showed that the dimer of glutaraldehyde (DGA) selectively damage the endothelial ability to regulate the vascular lumen during the changes of blood flow [43,44]. It would appear reasonable that MDA, a product of lipid peroxidation produced during oxidative stress, can also provoke endothelial dysfunction by degrading its ability to control the hydraulic resistance of blood vessels under the changes of shear stress applied to the vascular wall.

**Figure 7:**
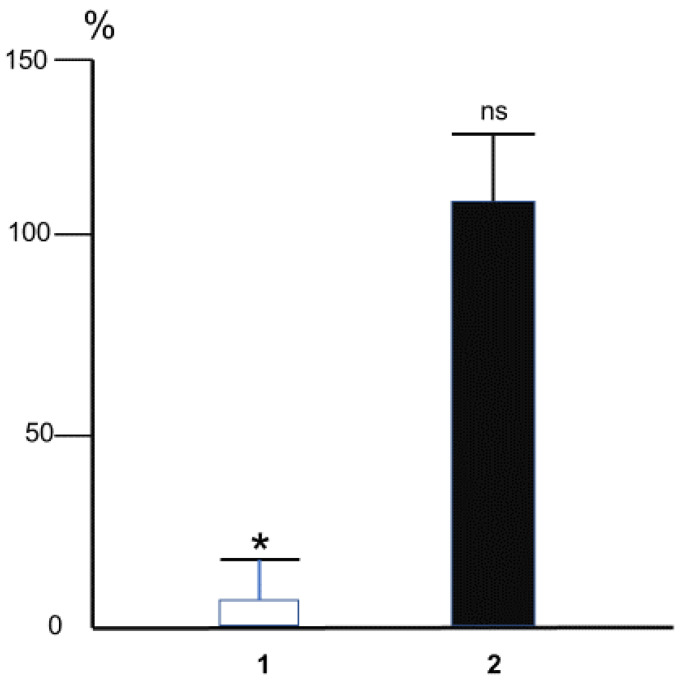
The effect of intra-arterial MDA (10 mM, 60 s) on changes of the hydraulic conductance of rat iliac artery (measured as the ratio of blood flow in the artery to the pressure drop on it) caused by overall triple step-like increase of blood flow (1) or acetylcholine (10^−6^ M) (2). The magnitude of change of arterial conductance at control conditions was taken for 100%. Bars correspond to the mean magnitudes of the dilator reactions rated in percentage to initial ones in each experiment. * *p* < 0.01, ns—not significant. *n* = 5 (Methods of the experiments were described in detail in [45]).

In experiments on anesthetized Wistar rats, we employed resistography to record the changes in hydraulic conductance of iliac artery in situ and corroborated the above hypothesis [45] by demonstrating that MDA injured the dilation of this artery in response to an increase in shear stress exerting virtually no effect on its dilation caused by acetylcholine (Figure 7). Logically, similar to DGA, MDA can selectively impair the mechanoreceptors in endotheliocytes formed by the EG layer [35,46,47,48,49] without affecting the potency of endothelial cells to relax the vascular smooth muscles in response to pharmacological dilators.

The endothelium-dependent control of vascular hydraulic resistance by the value of blood flow plays a key role in circulation. This property results from the potency of glycocalyx and endotheliocytes to react to the changes in shear stress applied to the vascular wall by the viscous friction of circulating blood [50,51]. It provides maximal blood flow during active hyperemia [44], down-regulates the constrictor reactions thereby preventing the development of vascular spasm [52,53], underlies the acute stage of collateral circulation development during occlusion of the major arteries [44], and adaptation of arterial resistance to the changes in blood viscosity [51]. Evidently, the degraded ability of glycocalyx to deform under the action of shear stress would provoke dysfunction of endothelium manifested by: (1) the loss of its potency to control the tone of vascular smooth muscles in response to the changes in blood flow velocity, and (2) the development of some pathological states such as arterial spasm, arterial hypertension, poor blood flow in organs and tissues, etc.

Especially important is the fact that the thickness of EG depends on shear stress: the greater the stress, the thicker the glycocalyx layer [33]. Therefore, the areas of the vessel wall exposed to low shear stress have thinner glycocalyx fraught with appearance of the loci with characteristic signs of atherosclerotic lesions that form the consequential atheromatous plaques [54]. This observation substantiates the hypothesis that EG is the barrier preventing penetration of atherogenic LDL into subendothelial space at the vascular wall [55]. Thinning of EG is secondary to decreased contents of hyaluronan and heparan sulfate, its major structural components [56] whose biosynthesis by endotheliocytes is decelerated when the shear stress is small [57].

Glycocalyx protects the endothelium against a moiety of damaging agents, including ROS [34,58]. Degradation of glycosaminoglycans in EG can be provoked by ROS generated by stimulated polymorphonuclear leukocytes [59] as well as by ROS produced during local or total ischemia/reperfusion injury [34,60,61]. The detrimental effect of such injury on the glycocalyx can be ameliorated by suppression of oxidative burst (rapid release of superoxide anions O_2_^•−^), evidently attesting to the leading role of ROS in destruction of the glycocalyx during ischemia/reperfusion [61]. Oxidized LDL administered to the animals decreases the thickness of glycocalyx, and this effect is absent in the presence of SOD and catalase [62,63]. Importantly, the oxidized LDL were routinely produced in these experiments [64,65] by free radical peroxidation of LDL in the presence of copper ions for 6 h [62,63]. In this oxidative reaction, not only oxidized (LOOH-containing) LDL was produced, but MDA and related MDA-modified LDL were also generated [66] (Figure 2). Further, LOOH can be subjected to homolytic cleavage producing the active alkoxy radicals LO^•^ capable of inducing free radical oxidation of organic substrates with production of O_2_^•−^ [66,67], which explains the reduction of the lesions to the glycocalyx in the presence of SOD and catalase [62,63]. Although these experiments cannot be unambiguously interpreted, they overall confirm implication of the free radical processes in the lesion to the endothelial glycocalyx. Importantly, the oxidatively modified LDL (probably MDA-LDL) can trigger thrombogenesis by up-regulating adhesion of the platelets [68] and monocytes [69,70] at the endothelium. Adhesion of polymorphonuclear neutrophils at the coronary endothelium is the pivotal event in ischemia/reperfusion injury [71]. Essentially, maintenance of the glycocalyx structure impedes the postischemic adhesion of the neutrophils [71].

The obtained results on laboratory animals show that MDA-LDL is quickly eliminated from blood and its disposal probably occurs in the liver [72]. Nevertheless, the described physiological effects of MDA and MDA-modified LDL can occur in case of a local increase in their levels. Therefore, preservation of the glycocalyx which protects the outer membrane of endotheliocytes depends largely on the presence and degree of oxidative stress. At the same time, breaking the protective layer of the glycocalyx [59,60] and destruction of endotheliocytes as a result of apoptosis probably make it easier for MDA-LDL laden with lipids to penetrate vascular walls, where they are involved in “foam cell” formation and development of primary pre-atherosclerotic (lipoidosis) damage.

## 6. Free Radical Peroxidation of Lipids and Preventive Treatment for Atherosclerosis: From Identifying Molecular Mechanisms of Atherogenesis to Justification for New Approaches to Pharmacotherapy

Based on the above data, naturally, we can assume that the non-toxic natural antioxidants can be used to suppress LDL free radical peroxidation. To implement this, numerous attempts were made to use safe natural antioxidants, such as α-tocopherol (vitamin E), for treatment of cardiac episodes. However, clinical trials were quite unsuccessful [3,17]. When the design of these trials is analyzed, it is surprising that α-tocopherol was used to inhibit LDL lipoperoxidation, while in numerous studies it was shown that α-tocopherol is not effective in suppressing LDL peroxidation both in vitro [73,74] and in vivo [75,76]. Moreover, it was demonstrated that there is another natural substance which provides effective protection of LDL from free radical peroxidation in vivo: coenzyme Q_10_ (we mean its reduced phenol form) [73,74,75,76]. The share of coenzyme Q_10_ in LDL is extremely small (no more than 2–3 molecules per particle which consists of about 650 phospholipid molecules) [77]. It is obvious that the protective effect of coenzyme Q_10_ in LDL can be realized only in the presence of an effective system for its reduction (bioregeneration). Currently, it is unclear how bioregeneration of coenzyme Q_10_ in LDL can occur. However, it should not be ruled out that free radical transformations of α-tocopherol and ascorbate play a certain role in this process [78,79].

It should be noted that some synthetic non-toxic antioxidants, such as probucol, can also effectively inhibit LDL lipoperoxidation [80,81,82,83]. Ultimately, research into the capability of biguanides [84,85], imidazole-containing peptides [86] and their derivatives [87] to bind and neutralize aggressive carbonyl compounds formed as secondary lipoperoxidation products and cause atherogenic modification of LDL is definitely promising. Therefore, preventive pharmacological correction of oxidative stress is also promising, but a radical solution has not been found yet, and it is a relevant issue in modern cardiology.

## 7. Conclusions

The mini review is devoted to the consideration of oxidative transformations of LDL and the role of free radical modifications of LDL in the molecular mechanisms of atherogenesis and endothelial dysfunction. The “atherogenicity” of the carbonyl modification of LDL is postulated by the secondary product of free radical peroxidation of unsaturated lipids with malondialdehyde (MDA). Based on the literature and proprietary data, we suggested that MDA-modified LDL can stimulate the expression of LOX-1 receptor biosynthesis and NADPH oxidase in endotheliocytes, which contributes to the development of endothelial dysfunction. Approaches to preventive pharmacotherapy of atherosclerosis are considered.
